# Adherent Placenta—Case

**Published:** 1875-08

**Authors:** B. R. Rives

**Affiliations:** Adams’ Station, Ga.


### ADHERENT PLACENTA—Case.

By B. R. RIVES, M.D., of Adams' Station, Ga.

A rare, if not a strange, case of retained placenta from morbid adhesion, with irregular contraction of the uterus, following abortion. After abortion, or labor at full term, from one to one and a half hours is generally the estimated interval that should elapse before the accoucheur is called on, by manual interference, to deliver the secundines. The plan, however, of our action has been not to be governed by any fixed rule but the one of remembering that safety depends upon its removal, and if it is not accomplished spontaneously at the time, or early after the expulsion of the fœtus, we proceed, as soon as the condition of the patient will admit of it, to its extraction. This then being a word as to our understanding and course of proceeding, the following case of abortion with retained and non-delivered placenta, not followed by any lochial discharge, or any whatever, showing that decomposition had taken place, neither by inflammation, or any other trouble as a sequence from retained placenta, is rare, if not strange, and it is for this that we have seen fit to give it this notice.

M., col., age forty, about five months advanced in pregnancy, had been sick with fever, pain in the right side, and cough for a week previous to the night of January 14th. When labor pains came on, and about day, having, as she supposed, a call to empty the bowels, to her surprise, dropped the fœtus in the chamber. Her condition some ten hours afterwards was the cause of our visit. We found her in the following condition: fever, pain and cough, as above stated; pulse 110; temperature not taken; tongue covered with a dark, thick and heavy coat; bowels constipated, with some tenderness on pressure; cough accompanied with the semi-transparent and adhesive expectoration so characteristic of pneumonitis. Learning that she had aborted, as stated, and that the placenta was retained, on making an examination we found the cord between the thighs, which parted on traction being made to trace it. After the necessary preparation for extraction, and upon introducing the hand, we learned that we had not only a retained placenta, but a clear case of hour-glass contraction, with the after-birth in the upper chamber of the uterus to manage. Finding the constricted ring firm and only large enough to admit the tip of the index finger, and taking into consideration the duration of the pregnancy, together with the interval that had elapsed from the time of the accident to our visit, we readily inferred more than the usual difficulties to overcome, and as there was no hemorrhage, withdrew the hand to use morphine hypodermically. Just here—being somewhat an admirer of the little instrument—I would take occasion to remark that I have heard of more than one case of fatal hemorrhage before the expulsion of the after-birth, which I have been inclined to attribute to the use of the syringe and morphia, and in ordinary cases of retention would add a word of caution against their wholesale use. In irregular contractions, however, the liability to hemorrhage is not so imminent, and in our own case the firmness of the ring demanded its use. But to resume. Taking advantage of the use of the syringe, and reintroducing the hand, we found that it passed readily into the lower chamber of the uterus, but failed, after the best directed effort, to pass the constriction with more than one finger. We learned enough, however, to know that there was morbid adhesion of the entire mass, and the best, and all that we could do, was to steady the uterine organ with the left hand externally, and separate, break through and remove such portions of the placenta as we could, which amounted to barely one-third of the entire mass. The other two-thirds yet remain; at least there was not during the puerperal month any lochial, sanguinous or putresent discharge, or any other of any kind whatever showing how it had been disposed of; neither did she suffer by the retention, and at this time is regular in her catamenia.

I know that Blundell and other authors of weight counsel the profession not to make much effort to remove it, provided the placenta break under the hand, yet others of equally as great weight advise the removal of the last particle, and I have, as nearly as possible, followed their advice. Those holding the former position think that more danger would accrue by attempting to separate the strongly-adherent portion than by leaving a part of it behind; but no one has pretended to think there was safety with any considerable portion of the placenta remaining.

It is worthy of remark that this woman informs me that she has had eleven children, and that after the expulsion of the after-birth she has not at any time been troubled with after-pains or lochial discharges. After learning the fact just stated, I determined, if she lived—which I thought extremely doubtful at the time—to make other inquiry as to the fact, not doubting but that in this instance there would be a departure from the rule peculiar to herself. The result was more than a confirmation of her statement, because, with the amount of retained placenta mentioned, there was no discharge, and I am abundantly confident that the amount left as stated is not over-estimated. After this fruitless effort, our patient being well nigh exhausted, and having learned that for the last five or six days she had suffered with fever, cough and pain in right side, with but little or no rest or sleep, I directed opium to be given her in grain doses every two hours, to rub the chest and bowels with turpentine every three hours, and after each application to cover the chest, after the custom of the Bellevue Hospital, with oil-cloth, urging at the same time the necessity for giving chicken water or soup as much as she could be prevailed upon to take without disturbing her rest, and brandy if necessary; but, should there be any hemorrhage, to use cold cloths to the bowels and give me notice.

15th, 8 o'clock A.M.—Had rested and slept some; cough and pain in side somewhat abated; pulse the same, but softer; some thirst; no after pains, no discharge per vaginam; not more than the usual tenderness over bowels, and as they had not moved, ordered an injection, and directed a continuance of treatment, adding fifteen grains of quinine in five-grain doses every two hours, commencing at sun-up on the 16th.

The case gradually improved from day to day under the use of opium, ipecac, turpentine, and oil-cloth for a week, at which time she was discharged as out of danger, and at this time, June 9th, is enjoying her usual good health.

That there have been cases of retention of part of the placenta, which, on becoming organized, forms a fleshy mass known as a mole, or that the chorion sometimes undergoes a peculiar metamorphosis which receives the appellation of uterine hydatids, or that the mass may possibly be absorbed, is not denied; but for a retained and disrupted placenta to be so completely absorbed as to prevent any after-pains, any lochial or fetid discharge, or any hemorrhage, neither to be followed by any increase in the already existing fever, or inflammation, or any departure from the usual getting up as a sequence of retention, does seem so remarkable that I should even feel disposed to distrust myself were it not that the facts were put beyond the possibility of a doubt. I know that in some cases an existing fever is apparently arrested by abortion, and that after still-born and putrid infants the lochial discharge is continued only for a few days, but this is not the case when the placenta is retained. That the placenta may remain and afterwards be mistaken for cancer, or the patient may die months afterwards from septicæmia, is admitted; in fact, I have seen, but cannot just now refer to, some reports of such cases; yet their after history was certainly different from this case.

The question, then, that presents itself to my mind is, what has become of the thing? All authority that I have been able to consult say that moles and hydatids are more commonly the result of retention than that of absorption, and clinically demonstrated the fact. While this may be thus clearly proven, I am disposed to think it not a verity, and inclined to the opinion, at least in this instance, that it has been absorbed. Nature has provided but two ways whereby she may rid herself of the secundines: one is by expulsion, and the other by absorption, and I see no reason why she should depart from her course to form moles, etc. It must also be remembered that this woman had passed the third month of her pregnancy, that the placenta was formed, that the villi of the chorion not engaged in its development had become atrophied, and hence it is not likely that such a degeneration would take place, and that if such had been the case it is highly probable there would be soon symptoms announcing the fact, such as uneasiness about the hypogastric region, some weight or pain about the uterus, menorrhagia, or some constitutional disturbance. It would also appear that the great rapidity with which absorption takes place under such circumstances is amply provided for by the admirable arrangements of nature in supplying its parts so abundantly with blood-vessels. It occurs to me that the proximity of the fœtal circulation to that of the mother is so nearly connected, if not, as some physiologists contend, direct, that so soon as the circulation is arrested by ligation absorption commences; or if this circulation is carried on without disturbing the molecular coherence of the vascular tissue or placental cell-wall, which may be increased by their enlargement and otherwise thining of their coats, the process would be facilitated when the placenta is firmly adherent. The patulous condition of the blood-vessels after parturition, and the enlarged and stimulated lymphatics that accompany them, seem to take an important part in the great work of repair; and if moles or hydatids be formed either before or after parturition, there would seem to have been some traumatic interference lessening the quantity or quality of nutrient supply. But again, the decidua is but the mucous membrane of the uterus enlarged and thickened, which with the glass is seen to be studded with minute openings—the mouths of follicles—some with simple unbranched tubes, and others with several branches with a capillary network around the glands or follicles, all taken together being that of which the placenta is formed, and when they have served their purpose of formation leaves a large area for absorption. But it may be said that the blood in the womb is poisonous; if so it is nevertheless homogeneous.

We then are inclined to think moles and hydatids not the rule but the exception, and more especially as some claim that the true hydatids—that is, cysts due to the presence of the acephalocyst—are rarely met with in the uterus. Neither do we think the is idea weakened by the experiments of Hildebrant with ergot to promote the absorption of uterine tumors. He, by the use of ergot, cuts off the nutritive supply upon which the tumor depends for its growth, and awaits the process of fatty degeneration that absorption may take place.

